# The Design and Characterization of a Flexible Tactile Sensing Array for Robot Skin

**DOI:** 10.3390/s16122001

**Published:** 2016-11-25

**Authors:** Zhangping Ji, Hui Zhu, Huicong Liu, Nan Liu, Tao Chen, Zhan Yang, Lining Sun

**Affiliations:** School of Mechanical and Electric Engineering, Collaborative Innovation Center of Suzhou Nano Science and Technology, Jiangsu Provincial Key Laboratory of Advanced Robotics, Soochow University, Suzhou 215000, China; jizhangping@foxmail.com (Z.J.); t_zhuhui@suda.edu.cn (H.Z.); Liunan@suda.edu.cn (N.L.); chent@suda.edu.cn (T.C.); yangzhan@suda.edu.cn (Z.Y.); lnsun@suda.edu.cn (L.S.)

**Keywords:** flexible electronics, capacitive tactile sensing array, robot skin, robotic obstacle avoidance

## Abstract

In this study, a flexible tactile sensing array based on a capacitive mechanism was designed, fabricated, and characterized for sensitive robot skin. A device with 8 × 8 sensing units was composed of top and bottom flexible polyethyleneterephthalate (PET) substrates with copper (Cu) electrodes, a polydimethylsiloxane (PDMS) dielectric layer, and a bump contact layer. Four types of microstructures (i.e., pyramids and V-shape grooves) atop a PDMS dielectric layer were well-designed and fabricated to enhance tactile sensitivity. The optimal sensing unit achieved a high sensitivity of 35.9%/N in a force range of 0–1 N. By incorporating a tactile feedback control system, the flexible sensing array as the sensitive skin of a robotic manipulator demonstrated a potential capability of robotic obstacle avoidance.

## 1. Introduction

In the past few decades, the development of intelligent robots has received increasing attention in research, industry, and family life [1]. Intelligent robot skin with tactile sensing capability can help robots operate in unknown environments and safely interact with people and objects [2]. In the literature, flexible tactile sensors can be classified into several sensing mechanisms, which are capacitive [3,4,5,6], piezoresistive [7,8,9,10], piezoelectric [11,12,13], thermoelectric [14], triboelectric [15,16], and other functional materials with sensing characteristics [17,18,19,20]. Among these, a capacitive sensing mechanism is usually preferred because of its simple structure, stable performance, and temperature independence [5]. Researchers from the University of Cambridge [21] presented a typical capacitive sensor, consisting of top and bottom gold electrodes and a silicone rubber dielectric layer. When an external force was applied, the distance between the gold electrodes decreased and the capacitance increased accordingly. The pressure sensitivity of the device was 1.4%/N at most, which was relatively low for a practical application.

One approach to increasing the sensitivity of the flexible tactile sensors was to utilize softer materials serving as the dielectric layer. For example, Lee et al. [22] demonstrated a capacitive tactile sensor using air as the dielectric layer. It was much easier to deform under a small force and thus exhibited high sensitivity. A sensitivity of 3%/mN was measured for a small deflection. However, the operating range was limited because the air gap disappeared quickly under little force. Another way to increase the sensitivity is to incorporate microstructures on the surface of the dielectric layers. Liang et al. [23] presented a flexible capacitive tactile sensor embedded with a polydimethylsiloxane (PDMS) pyramid array as a dielectric layer, which greatly enhances the sensitivity up to 67.2%/N. It is known that a different microstructure morphology and geometry can lead to a different sensitivity of the tactile sensor; however, there are few articles in which the variation is discussed in detail. In this work, we designed four different types of microstructures on the dielectric layer and compared their static characteristics so as to gain an improved performance for capacitive sensing.

For practical applications, various tactile sensors have been demonstrated as having promising pressure sensing properties in robot skin [24,25,26,27]. By referring to the human sense of touch, Crowder [28] listed specifications of a tactile sensor suitable for robot skin applications, such as a force range of 0.4–10 N, and spatial resolution of 1–2 mm. Similarly, Dahiya and Dario [29,30,31,32] reported design parameters for a robotic tactile system, which included human-like spatial sensitivity viz. 1 mm (fingers) and 5 mm (palm), sensitivity to forces spanning from 0.01 N to 10 N, and an incremental force resolution of 0.01 N. In most cases, a signal processing circuit is required as the interface between the tactile sensor and the robot controller. Mei and his research group mounted their flexible tactile sensor on a prosthetic hand to measure the grasping force [24]. A typical signal processing circuit, consisting of an analog digital conversion and a microprogrammed control unit (MCU) was employed. The researchers from the iCub Facility presented a robot skin tactile system on humanoid robots [25,26,27]. It incorporated distributed pressure sensors and a capacitance to a digital converter that measured the capacitance of each sensor. The robot skin was able to cover a large area of a robot body. However, there was hysteresis in the experiment, and the substrates of the sensor were not flexible enough.

In this work, we present an 8 × 8 flexible tactile sensing array as robot skin based on a capacitive sensing mechanism. Four microstructured elastomers as the dielectric layer were embedded, and their static characteristics were explored and are discussed here. Moreover, a tactile feedback system was built up and applied on a robotic manipulator to realize the function of robot obstacle avoidance.

## 2. Design and Fabrication

### 2.1. Design of the Tactile Sensing Array

**Figure 1:**
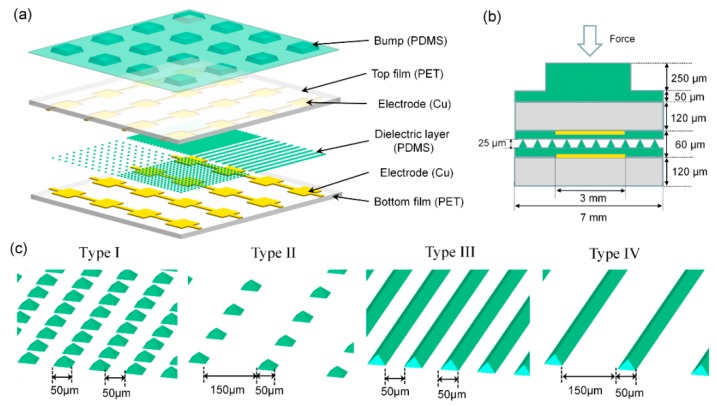
(**a**) Exploded view of the capacitive tactile sensing array. (**b**) Cross-section view of one sensing unit, and (**c**) schematic view of different geometries of the microstructures on the polydimethylsiloxane (PDMS) layer, including pyramids with spaces of 50 μm (type I) and 150 μm (type II), and V-shape grooves with spaces of 50 μm (type III) and 150 μm (type IV).

As illustrated in Figure 1a, the proposed tactile sensing array consisted of 8 × 8 capacitive sensing units with a spatial resolution of 7 mm. The sensing array was composed of top and bottom substrates patterned with Cu electrodes, a microstructured dielectric layer, and a bump contact layer. The top and bottom substrates were 120 µm thick and made of flexible polyethyleneterephthalate (PET) films. The top and bottom Cu electrodes were patterned on the PET substrates and vertically aligned to each other. A 60-µm-thick PDMS film with microstructure patterns was selected as the dielectric layer due to its low Young’s modulus, high dielectric constant, and high structural flexibility. A PDMS bump contact layer with 300 µm in height was used to concentrate the force intensity. Figure 1b shows a cross-sectional view of a single capacitive sensing unit. When an external force was applied on the bump, the dielectric layer deformed, which indicated a gap decrease between the top and bottom electrodes of the capacitor. Accordingly, the capacitance would be increased. Such capacitance variation was used to calculate the applied tactile force quantitatively.

Compared with an unstructured dielectric layer, a microstructured dielectric layer of the same thickness has a higher tactile sensitivity [33]. In this design, the dielectric layer was divided into four parts with four different microstructure patterns, as shown in Figure 1c. Two of them were pyramid structures with the same critical feature size of 50 µm × 50 µm but with different feature spaces of 50 µm (type I) and 150 µm (type II), respectively. The other two were V-shape grooves 50 µm in width and 30 mm in length, where the feature spaces were 50 µm (type III) and 150 µm (type IV), respectively. All microstructures had a height of 25 µm. The variations of the microstructures were explored to optimize the tactile sensitivity.

### 2.2. Fabrication Process of the Tactile Sensing Array

**Figure 2:**
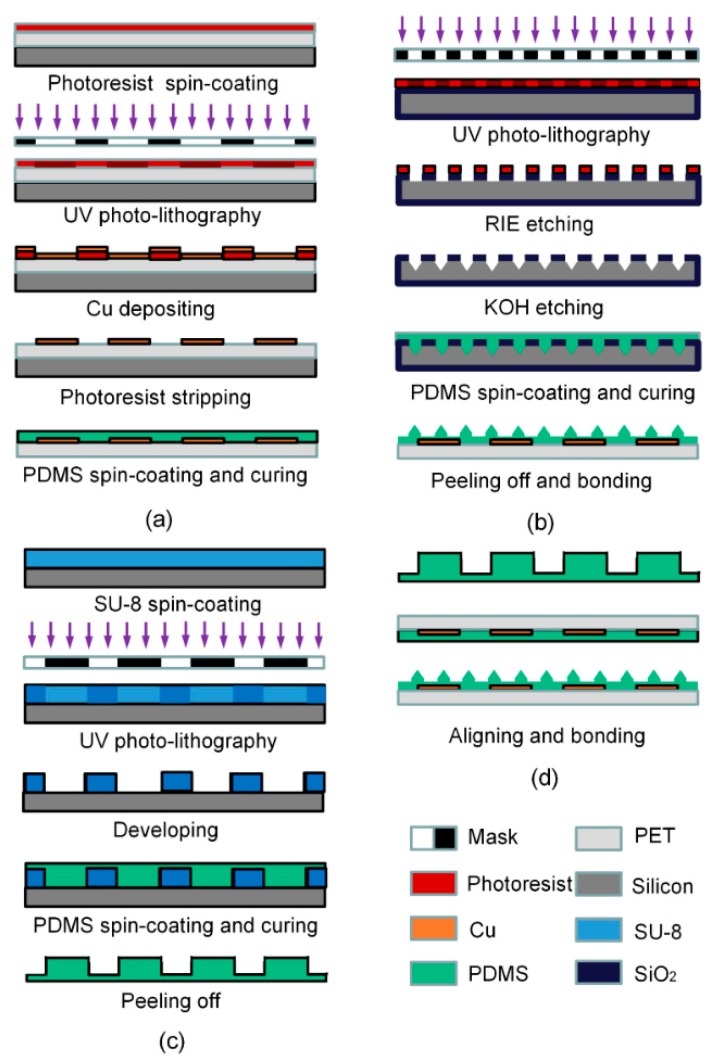
Fabrication process of the proposed tactile sensing array: (**a**) formation of the top and bottom electrodes on the polyethyleneterephthalate (PET) substrates; (**b**) formation of the microstructured dielectric layer; (**c**) formation of the bump contact layer, and (**d**) the bonding sequence.

The fabrication process flow of the tactile sensing array is illustrated in Figure 2. It includes four main procedures. The formation of the top and bottom electrodes on a PET substrate is shown in Figure 2a. A 120-μm-thick PET film was firstly mounted on a 4-inch silicon (Si) wafer, and a 1.5-μm-thick AZ5214 positive photoresist (PPR) was spin-coated on the PET film. This was followed by UV photo-lithography and a developing process to obtain the patterned PPR. After plasma treatment on the top surface, 30-nm-thick titanium (Ti) and 400-nm-thick Cu layers were magnetron sputtered. Then, a photoresist stripping process was conducted by using an acetone solution, and thereafter the Cu electrodes were kept. After that, a 20-μm-thick PDMS was spin-coated to cover the patterned electrodes. After curing the PDMS film at 80 °C for 3 h, the PET film with patterned Cu electrodes was peeled off from the Si substrate.

The fabrication of the microstructured dielectric layer is critical for the tactile sensing array. The process flow is shown in Figure 2b. Firstly, a 500-nm-thick SiO_2_ layer was grown on a 400-μm-thick, 4-inch Si wafer. To form the microstructures, a PPR layer was spin-coated and exposed as a mask layer. Later on, the SiO_2_ layer was patterned by a reactive ion etching (RIE) method, serving as a hard mask. After the PPR was removed with an acetone solution, the Si wafer was immersed in a 30% KOH solution for 2.5 h. The anisotropic etching rate was about 10 μm/h. With the patterned SiO_2_ hard mask, pyramid grooves with side walls of an angle of 45° were formed. Therefore, the Si wafer with reversed microstructures served as a mold to transfer the various microstructures on the PDMS dielectric layer. After a surface treatment on the Si mold using mold releasing agents, a 40-µm-thick PDMS layer was spin-coated onto the Si mold. After a curing process at 80 °C for 3 h, the PDMS film with well-patterned microstructures was peeled off completely.

For the preparation of a bump contact layer, a negative photoresist (NPR) SU-8 was used as a transfer mold based on its good micro-machining and low surface energy characteristics, as shown in Figure 2c. Firstly, a 300-μm-thick SU-8 NPR was spin-coated on a 4-inch Si wafer and pre-baked at 95 °C for 1 h. After UV exposure, the SU-8 mold was again transferred onto the hot plate for post-baking. Afterwards, the SU-8 mold was completed after developing for 25 min and hard-baking for 30 min at 200 °C. The PDMS was spin-coated on the SU-8 mold. After curing treatment, the PDMS bump contact layer was peeled off from the SU-8 mold.

The fabricated top and bottom PET substrates with Cu electrodes, the microstructured dielectric layer, and the PDMS bump layer needed to be bonded together, as shown as in Figure 2d. The microstructured PDMS layer was firstly attached on the bottom PET substrates with Cu electrodes after being treated by oxygen plasma. Then, the PDMS layers on the top and bottom substrates were treated with oxygen plasma to improve their adhesive properties. Afterwards, we used a mask aligner for aligning and bonding, a process that included a moving platform and a microscope. Finally, the bump layer was aligned and attached to the top of the sensor array using a similar approach.

**Figure 3:**
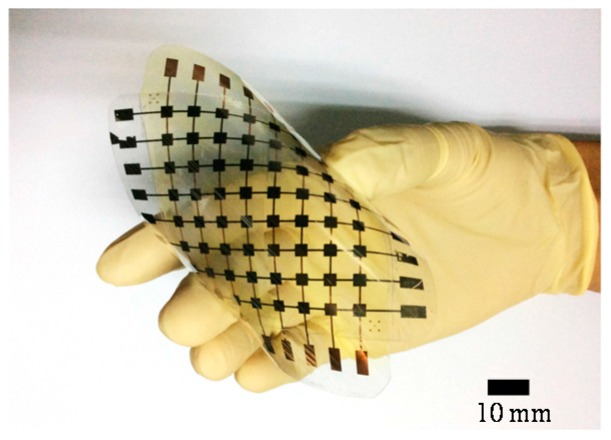
The fabricated capacitive sensing array bent by hand.

**Figure 4:**
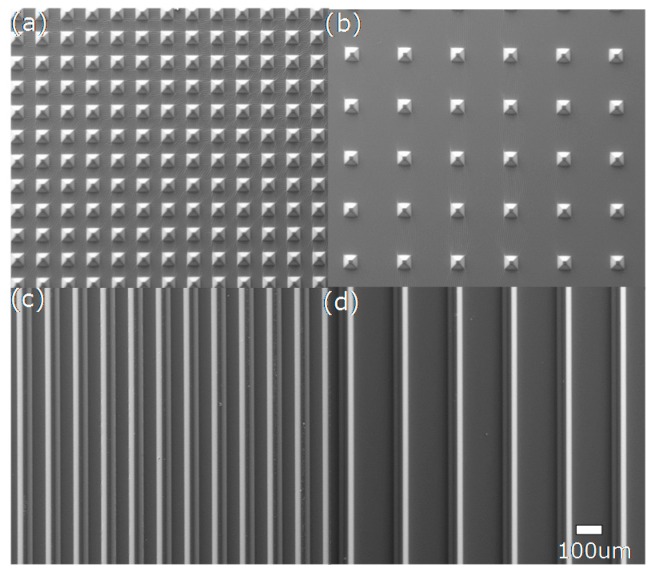
SEM images of four different microstructures on the PDMS layer: (**a**) type I; (**b**) type II; (**c**) type III; and (**d**) type IV.

Figure 3 shows the fabricated tactile sensing array with an overall dimension of 8 cm × 8 cm. It can be seen that the sensing array has good flexibility and can be easily bent by hand. Figure 4 shows the enlarged SEM images of the four different microstructures on the PDMS layer, which were well fabricated and consistent with the original design. The fabricated pyramid structures with different feature spaces of 50 µm (type I) and 150 µm (type II) are shown in Figure 4a,b, respectively. The other two fabricated V-shape grooves (type III and type IV) are shown in Figure 4c,d.

## 3. Static Characterization and Discussion

**Figure 5:**
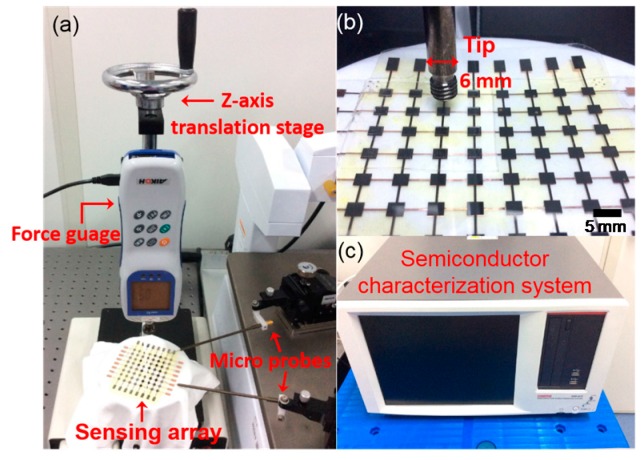
Measurement setup to study the sensing performance of the tactile sensing array: (**a**) the tactile sensing array placed on a plate with a force gauge above it; (**b**) a partially enlarged view of (**a**); and (**c**) the semiconductor characterization system.

To study the tactile sensing characteristics, a custom-made setup was constructed as shown in Figure 5a. The sensing array with 8 × 8 sensing units was placed under a force gauge. The force gauge is from Aikoh Engineering with a measurement range of 20 N. Its measuring precision is 0.2%. In order to apply a precisely pressure on each tactile sensing unit, the force gauge with an end effector of a diameter of 6 mm was fixed on a precision z-axis translation stage. Figure 5b shows the enlarged view of the sensing array and the end effector of the force gauge. A semiconductor characterization system (Keithley 4200-SCS, Tektronix, Beaverton, OR, USA) as shown in Figure 5c was used to measure the capacitance of each sensing unit under different force pressures, while there were two micro probes connecting the pads of the sensing unit and the semiconductor system.

In the experiment, four types of sensing units, i.e., types I–IV, with various PDMS microstructures were characterized. Because the characteristics of the capacitor units of one type of sensing array are similar, we selected only one typical capacitor unit of each type. When an increasing force between 0 and 10 N was applied onto one of the sensing units, the capacitance was measured for each increment. The sensitivity S of a capacitive sensor can be expressed as:

(1) $$S=\frac{\Delta C/C_{0}}{\Delta F}=\frac{1}{d_{0}}\cdot \frac{\Delta d}{\Delta F}$$

where *C_0_* is the initial capacitance, *d_0_* is the initial distance, *ΔC* is the relative change of capacitance, *ΔF* is the relative change of applied force, and *∆d* is the distance variation of the dielectric layer. It can be seen from this formula that the sensitivity *S* is in direct proportion to the deformation *∆d*.

**Figure 6:**
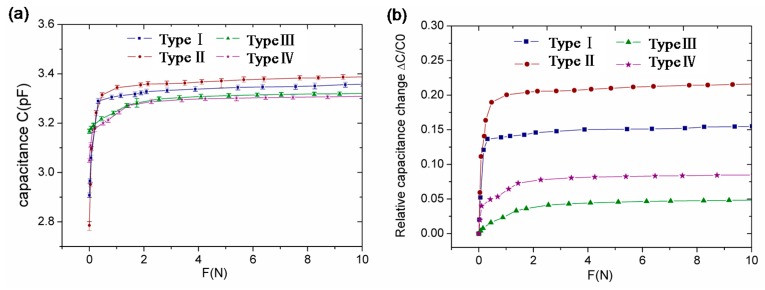
(**a**) The measured capacitance of the four fabricated units with different PDMS structures within the applied force of 10 N and (**b**) measured capacitance change of the four fabricated units with different PDMS structures within the applied force of 10 N.

Figure 6a presents the measured capacitance of four different sensing units as a function of applied force. The error bar denotes the maximum and minimum values, which are small enough to be neglected. The initial capacitance of the four types of sensing units were approximately the same, which were 2.91 pF for type I, 2.79 pF for type II, 3.16 pF for type III, and 3.05 pF for type IV. Based on the measured data in Figure 6a, the relative change of capacitance (*ΔC/C_0_*) as a function of applied force (F) can be obtained as shown in Figure 6b. It can be seen that the sensitivity of a capacitive sensing unit varies between different microstructured PDMS dielectric layers. The sensitivities of the pyramid-structured units were higher than those of the V-shape-structured units. Within a force range of 0–1 N, the sensitivities of the pyramid-structured units were 15.2%/N and 35.9%/N for types I and II, respectively. These were one order of magnitude higher than those of the V-shape-structured units, which were 2.5%/N and 9.4%/N, respectively. The highest sensitivity of 35.9%/N was obtained from the sensing unit with a pyramid dielectric layer of 150 µm in space. When the applied force is more than 1 N, the rate of change of capacitance becomes very small and gradually reaches saturation. This is because the microstructures in the dielectric layer are completely pressed and the thickness reduction is only introduced by the compressive strain of the dielectric layer.

**Figure 7:**
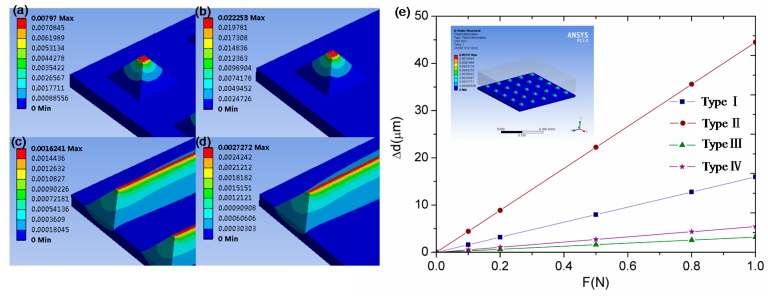
Strain distribution of the four different dielectric layers under a uniform force of 0.5 N: (**a**) type I; (**b**) type II; (**c**) type III; and (**d**) type IV; (**e**) deformation of the four different dielectric layers within the applied force of 1 N.

To verify this statement, finite element analysis was conducted by Ansys Workbench Software. In the model, four different microstructured PDMS elastomer were embedded as the dielectric layer. Young’s modulus of the PET and PDMS materials were 4 GPa and 5.5 MPa, separately. Poisson’s ratio of these two were set as 0.4 and 0.49, respectively. Figure 7a–d show the strain distribution of the four different dielectric layers under a uniform force of 0.5 N. Figure 7e shows the deformation of the four different dielectric layers within the applied force of 1 N. It can be seen that the deformations of pyramid structures of types I (space of 50 µm) and II (space of 150 µm) are higher than those of the V-shape structures of type III (space of 50 µm) and type IV (space of 150 µm). The dramatic increase in deformation of the pyramid structure over the V-shaped structure can be attributed to that the pyramid structure is easier to be compressed than the V-shaped structure. And it is because there is more air voids and less elastic resistance force in the pyramid structure. Similarly, the wider space between two adjacent microfeatures could lead to less elastic resistance force. Therefore, the deformation of type II with a space of 150 µm is higher than that of type I with a space of 50 µm under the same force.

We summarized the force range and sensitivity of those similar devices in the literature, and they are listed in Table 1. It can be seen that the force range and sensitivity of our sensing unit are higher than those most commonly reported. Although the sensitivity of tactile sensors presented in [3,23] are higher than ours, the corresponding force range is lower.

## 4. Tactile Feedback Experiment for Robot Obstacle Avoidance

**Figure 8:**
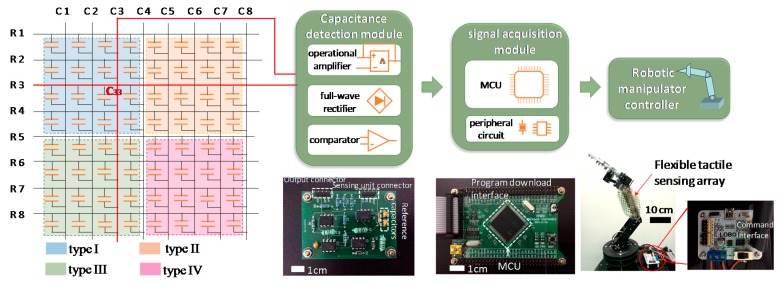
Schematic diagram of the tactile feedback system for robot obstacle avoidance. Photographs of three printed circuit boards correspond to the three modules above them respectively.

For the demonstration of robot obstacle avoidance, the fabricated 8 × 8 sensing array was employed as the tactile skin of a robotic manipulator. When an external force in an unconstructed environment is applied on an arbitrary sensing unit of the tactile skin, the working robot should be able to detect and respond quickly to avoid adverse consequences. A tactile feedback system was built as shown in Figure 8. The 8 × 8 tactile sensing array was considered as an array of capacitors. The capacitance change of a sensing unit can be detected by a capacitance detection module and transformed into voltage variation. The trigger voltage was sent to a signal acquisition system, and a program interrupt occurred. The interrupt command enabled the robotic manipulator to change its motion and avoid obstacles.

**Figure 9:**
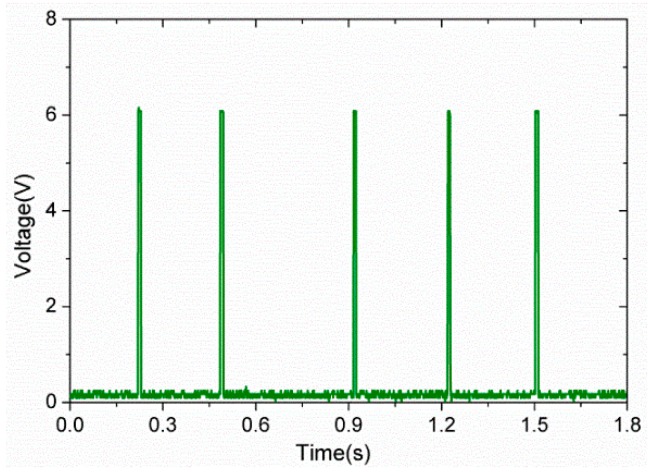
Output voltage from the capacitance detection module while applying force on the tactile sensing array via finger tapping.

The capacitance detection module consisted of an operational amplifier unit, a full-wave rectifier unit, and a comparator unit. The operational amplifier unit compared the capacitance of the sensing unit with a reference capacitor and enlarged the input/output signal. Then, the output AC signal was rectified by a full-wave rectifier module, and the obtained DC signal was connected to a comparator. If the DC voltage was above the reference voltage, the output signal would increase up to 6 V, which is equal to the value of the power supply of the comparator. Otherwise, the output was set to zero. As shown in Figure 9, when five random finger tappings were applied on one of the sensing units C33 (column 3 row 3) within 2 s, five pulse signals of about 6 V were produced through the capacitance detection module.

**Figure 10:**
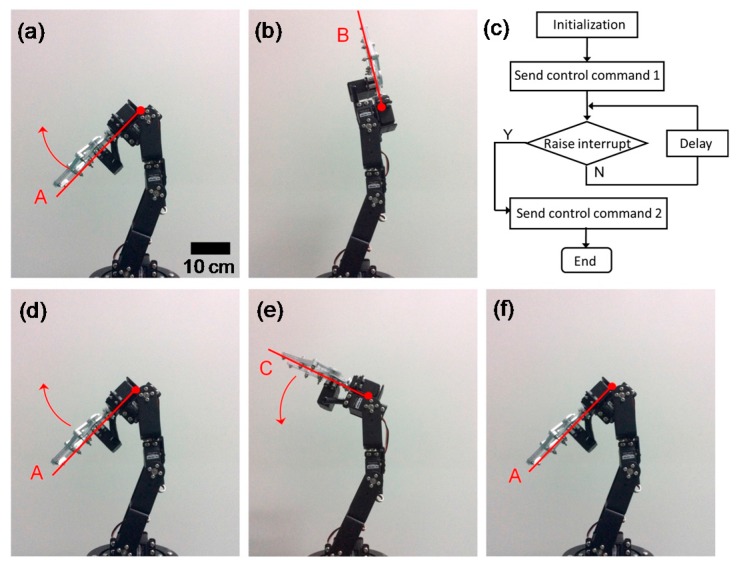
Experimental results of the tactile feedback system in the application of robot manipulator obstacle avoidance: (**a**,**b**) the movement of the forearm without interruption; (**c**) program flowchart of the MCU; (**d**–**f**) the movement of the forearm with force applied on the sensing array.

The printed circuit board integrated with the signal acquisition module is shown in Figure 8. The signal acquisition module connected to the controller of the robotic manipulator mainly consisted of a microprogrammed control unit (MCU) and a peripheral circuit, which included a crystal oscillator, a power module, and an AD convert module. The MCU was in charge of acquiring, processing, sending, and receiving data and commands. Figure 10c shows a program flowchart of the MCU. It started from an initialization of the system clock and serial ports. When the main program was sent to the controller, the robotic manipulator would move accordingly. At the same time, the interrupt program was on hold and waited for a call. When finger tapping was applied on the tactile sensing unit, which indicated that the robotic manipulator encountered an obstacle during the movement, a voltage pulse from the capacitance detection circuit would be transmitted to the MCU and trigger the interrupt program. While executing the interrupt service program, the control command enabled the robot to move back. After the robot returned to the defined position, the interrupt program was complete. The main program continued until the robotic manipulator stop running. The response time of the tactile feedback system is no more than 10 ms, which is fast enough for various applications.

The tactile feedback experiment was successfully carried out on a robot manipulator of six degrees of freedoms. Figure 9 shows the feedback sequences of its forearm movement, where Position A represents the initial state of the forearm and Position B denotes the end state. According to the main program, the forearm moved from position A (Figure 10a) to position B (Figure 10b) without interruption. However, during the process of the main program, once finger tapping force was applied on the sensing unit at position C, the interrupt program would be triggered and the forearm immediately moved back to its initial position A. This experiment of robot obstacle avoidance thus indicated that the tactile feedback of the flexible sensing array was very sensitive and effective.

## 5. Conclusions

In this study, we designed and fabricated a capacitive sensing array using microstructured PDMS as a dielectric layer to enhance the array’s sensitivity. The force sensing characteristics of the four different microstructured geometries provided the optimal pyramid structures with a space of 150 µm, which featured a higher sensitivity (35.9%/N). We also demonstrated a tactile feedback system for the application of robotic obstacle avoidance. The tactile sensing array on the robotic manipulator showed an effective and quick response in an unconstructed environment for the secure interaction of robot motion. In future work, the tactile sensing array needs to be optimized to increase the sensitivity, and the tactile feedback system needs to be enhanced with multipath control. The tactile sensing array attached on a robot arm will be developed for multi-directional obstacle avoidance.

## Figures and Tables

**Table 1:** Sensitivities of other tactile sensors in the literature.

Tactile Sensor	Dielectric Layer	Pressure Range	Sensitivity
Metzger [34]	Foam	0–0.1 N	2%/N
Shimojo [35]	Pressure conductive rubber	0–100 N	1.5%/N
Cotton [21]	PDMS	0–0.32 N	20%/N
Liang [23]	PDMS	0–0.5 N	67.2%/N
Petropoulos [3]	Air	0–0.6 N	57.4%/N
Lee [22]	Air	0–40 mN	3%/mN
Our work	PDMS	0–1 N	35.9%/N

## References

[1] Hall E.L. (2001). Intelligent robot trends and predictions for the net future. Proc. SPIE Int. Soc. Opt. Eng..

[2] Lee M.H., Nicholls H.R. (1999). Tactile sensing for mechatronics—A state of the art survey. Mechatronics.

[3] Petropoulos A., Kaltsas G., Goustouridis D., Gogolides E. (2009). A flexible capacitive device for pressure and tactile sensing. Sci. Direct Procedia Chem..

[4] Muhammad H.B., Oddo C.M., Beccai L., Recchiuto C., Anthony C.J., Adams M.J., Carrozza M.C., Hukins D.W.L., Ward M.C.L. (2011). Development of a bioinspired MEMS based capacitive tactile sensor for a robotic finger. Sens. Actuators A Phys..

[5] Maiolino P., Galantini F., Mastrogiovanni F., Gallone G., Cannata G., Carpi F. (2015). Soft dielectrics for capacitive sensing in robot skins: Performance of different elastomer types. Sens. Actuators A Phys..

[6] Kim H.K., Lee S., Yun K.S. (2011). Capacitive tactile sensor array for touch screen application. Sens. Actuators A Phys..

[7] Yao Y., Glisic B. (2015). Detection of steel fatigue cracks with strain sensing sheets based on large area electronics. Sensors.

[8] Lipomi D.J., Michael V., Benjamin T.C.K., Hellstrom S.L., Lee J.A., Fox C.H., Bao Z. (2011). Skin-like pressure and strain sensors based on transparent elastic films of carbon nanotubes. Nat. Nanotechnol..

[9] Pan L., Chortos A., Yu G., Wang Y., Isaacson S., Allen R., Shi Y., Dauskardt R., Bao Z. (2013). An ultra-sensitive resistive pressure sensor based on hollow-sphere microstructure induced elasticity in conducting polymer film. Nat. Commun..

[10] Cheng L., Cui Y.L., Tian G.L., Shu Y., Wang X.F., Tian H., Yang Y., Wei F., Ren T.L. (2015). Flexible CNT-array double helices Strain Sensor with high stretchability for Motion Capture. Sci. Rep..

[11] Kwi-Il P., Hwan S.J.H., Tae G., Jeong C.K., Ryu J., Koo M., Choi I., Lee S.H., Byun M., Wang Z.L. (2014). Highly-efficient, flexible piezoelectric PZT thin film nanogenerator on plastic substrates. Adv. Mater..

[12] Kobayashi T., Yamashita T., Makimoto N., Takamatsu S., Itoh T. Ultra-thin piezoelectric strain sensor 5 × 5 array integrated on flexible printed circuit for structural health monitoring by 2D dynamic strain sensing. Proceedings of the IEEE 29th International Conference on Micro Electro Mechanical Systems (MEMS).

[13] Dagdeviren C., Su Y., Joe P., Yona R., Liu Y., Kim Y.S., Huang Y., Damadoran A.R., Xia J., Martin L.W. (2014). Conformable amplified lead zirconate titanate sensors with enhanced piezoelectric response for cutaneous pressure monitoring. Nat. Commun..

[14] Rotzetter A.C.C., Schumacher C.M., Bubenhofer S.B., Grass R.N., Gerber L.C., Zeltner M., Stark W.J. (2012). Thermoresponsive polymer induced sweating surfaces as an efficient way to passively cool buildings. Adv. Mater..

[15] Dhakar L., Pitchappa P., Tay F.E.H., Lee C. (2015). An intelligent skin based self-powered finger motion sensor integrated with triboelectric nanogenerator. Nano Energy.

[16] Dhakar L., Gudla S., Shan X., Wang Z., Tay F.E.H., Heng C.-H., Lee C. (2016). Large scale triboelectric nanogenerator and self-powered pressure sensor array using low cost roll-to-roll UV embossing. Sci. Rep..

[17] Lee C., Jug L., Meng E. (2013). High strain biocompatible polydimethylsiloxane-based conductive graphene and multiwalled carbon nanotube nanocomposite strain sensors. Appl. Phys. Lett..

[18] Pang C., Lee G.Y., Kim T.I., Kim S.M., Kim H.N., Ahn S.H., Suh K.Y. (2012). A flexible and highly sensitive strain-gauge sensor using reversible interlocking of nanofibers. Nat. Mater..

[19] Xiao X., Yuan L., Zhong J., Ding T., Liu Y., Cai Z., Rong Y., Han H., Zhou J., Wang Z.L. (2011). High-strain sensors based on ZnO nanowire/polystyrene hybridized flexible films. Adv. Mater..

[20] Wang C., Hwang D., Yu Z., Takei K., Park J., Chen T., Ma B., Javey A. (2013). User-interactive electronic skin for instantaneous pressure visualization. Nat. Mater..

[21] Cotton D.P.J., Graz I.M., Lacour S.P. (2009). A multifunctional capacitive sensor for stretchable electronic skins. IEEE Sens. J..

[22] Lee H.K., Chang S.I., Yoon E. (2006). A flexible polymer tactile Sensor: Fabrication and modular expandability for large area deployment. J. Microelectromech. Syst..

[23] Liang G., Wang Y., Mei D., Xi K., Chen Z. (2015). Flexible capacitive tactile sensor array with truncated pyramids as dielectric layer for three-axis force measurement. J. Microelectromech. Syst..

[24] Wang Y., Liang G., Mei D., Zhu L., Chen Z. A flexible capacitive tactile sensor array with high scanning speed for distributed contact force measurements. Proceedings of the IEEE 29th International Conference on Micro Electro Mechanical Systems (MEMS).

[25] Maiolino P., Maggiali M., Cannata G., Metta G., Natale L. (2014). A flexible and robust large scale capacitive tactile system for robots. IEEE Sens. J..

[26] Cannata G., Maggiali M., Metta G., Sandini G. An embedded artificial skin for humanoid robots. Proceedings of the IEEE International Conference on Multisensor Fusion and Integration for Intelligent Systems.

[27] Maiolino P., Ascia A., Maggiali M., Natale L., Cannata G., Metta G., Berselli G. (2012). Large scale capacitive skin for robots. Smart Actuation and Sensing Systems—Recent Advances and Future Challenges.

[28] Crowder R.M. Automation and Robotics. http://www.soton.ac.uk/~rmc1/robotics/artactile.htm.

[29] Dario P., Rossi D. (1985). Tactile sensors and gripping challenge. IEEE Spectr..

[30] Howe R.D. (1994). Tactile sensing and control of robotics manipulation. J. Adv. Robot..

[31] Dahiya R.S., Metta G. (2010). Tactile sensing: From humans to humanoids. IEEE Trans. Robot..

[32] Dahiya R.S., Valle M. Tactile sensor arrays for humanoid robot. Proceedings of the 3rd International Conference on PhD Research in Microelectronics and Electronics.

[33] Tee C.K., Chortos A., Dunn R.R., Schwartz G., Eason E., Bao Z. (2014). Tunable flexible pressure sensors using microstructured elastomer geometries for intuitive electronics. Adv. Funct. Mater..

[34] Metzger C., Fleisch E., Meyer J. (2008). Flexible-foam-based capacitive sensor arrays for object detection at low cost. Appl. Phys. Lett..

[35] Shimojo M., Namiki A., Ishikawa M. (2004). A tactile sensor sheet using pressure conductive rubber with electrical-wires stitched method. IEEE Sens. J..

